# Familial MEN1 Syndrome with Atypical Renal Features and a Coexisting CLDN16 Variant: A Case Series

**DOI:** 10.3390/jcm14155447

**Published:** 2025-08-02

**Authors:** Ioannis Petrakis, Eleni Drosataki, Dimitra Lygerou, Andreas Antonakis, Konstantina Kydonaki, Marinos Mitrakos, Christos Pleros, Maria Sfakiotaki, Paraskevi Xekouki, Kostas Stylianou

**Affiliations:** 1 Department of Nephrology, University of Crete, Voutes, 70500 Heraklion, Crete, Greece; petrakgia@gmail.com (I.P.); elenidro2@hotmail.com (E.D.); dimitra.ligerou@gmail.com (D.L.); antonakisandreas@yahoo.gr (A.A.); k.ntina@hotmail.com (K.K.); marmit90@hotmail.com (M.M.); xpleros@gmail.com (C.P.); 2 Department of Endocrinology, University of Crete, Voutes, 70500 Heraklion, Crete, Greece; mariasfak@yahoo.gr (M.S.); pxekouki@uoc.gr (P.X.)

**Keywords:** MEN1, nephrocalcinosis, hypomagnesemia

## Abstract

**Background and Clinical Significance:** Multiple Endocrine Neoplasia type 1 (MEN1) is a rare autosomal dominant disorder caused by mutations in the MEN1 gene. Although primarily characterized by endocrine tumors, renal manifestations remain underreported. **Case Presentation:** We report a three-generation family carrying a pathogenic *MEN1* mutation (c.1351-3_1359del) with a co-occurring *Claudin 16* (*CLDN16*) variant (c.324+13C>G). Genetic testing included MLPA and whole-exome sequencing (WES), with bioinformatics analysis validating variant pathogenicity. All three patients exhibited primary hyperparathyroidism, hypercalcemia, hypercalciuria, early nephrocalcinosis, and renal hypomagnesemia. The CLDN16 variant, previously considered benign, co-segregated with hypomagnesemia and renal involvement, suggesting a potential modifying role. **Conclusions:** These findings support the need for comprehensive genetic screening in MEN1 patients with atypical renal presentations. Concomitant genetic variations can alter the principal phenotype.

## 1. Introduction and Clinical Significance

Multiple Endocrine Neoplasia (MEN) refers to a group of rare, inherited syndromes characterized by the development of tumors in multiple endocrine glands [1]. The most common form, MEN1, is caused by mutations in the *MEN1* gene and is associated with tumors in the parathyroid glands, enteropancreatic neuroendocrine system, and pituitary gland [2]. MEN2, caused by mutations in the RET proto-oncogene, leads to endocrine tumors, including medullary thyroid carcinoma (MTC), pheochromocytomas, and parathyroid hyperplasia. MEN2 is further classified into MEN2A, MEN2B with extra-endocrine features, and familial medullary thyroid carcinoma [3]. *MEN1* is a tumor suppressor gene located on chromosome 11q13, which encodes menin, a nuclear scaffold protein involved in gene transcription, tumor suppression, cell cycle regulation, genomic stability, and Wnt/β-catenin signaling [4,5]. More than 1000 *MEN1* pathogenic variants have been identified, with the majority (70%) predicting premature truncation or loss of the menin protein due to frameshift, nonsense, or splice-site variants [6,7,8,9,10,11]. MEN1 follows an autosomal dominant inheritance pattern and manifests primarily as primary hyperparathyroidism (PHPT), gastroenteropancreatic neuroendocrine tumors (GEP-NETs), and pituitary adenomas [12]. In some cases, MEN1 is also associated with other neoplasms, such as bronchopulmonary and thymic NETs, adrenal tumors (primarily adenomas but occasionally carcinomas), angiofibromas, lipomas, and thymic carcinoid tumors [13]. The presence of MEN1 is clinically defined as the occurrence of two or more primary MEN1 tumor types, or in family members of a patient with a clinical diagnosis of MEN1, the occurrence of one of the MEN1-associated tumors [14].

While MEN1 primarily affects endocrine tissues, its impact on renal function remains underexplored. Hyperparathyroidism-induced hypercalcemia has been implicated in renal complications, including nephrocalcinosis, nephrolithiasis, polyuria, and progressive kidney dysfunction, all of which may contribute to increased morbidity and mortality [15,16]. However, the role of genetic modifiers in renal dysfunction within MEN1 patients has not been well studied.

Claudin-16 is a tight junction protein expressed in the thick ascending limb of the loop of Henle and plays a crucial role in paracellular calcium and magnesium reabsorption [17,18]. It is encoded by the *CLDN16* gene, which is located on chromosome 3q27 [19]. Mutations in *CLDN16* are associated with familial hypomagnesemia with hypercalciuria and nephrocalcinosis (FHHNC), a disorder characterized by renal magnesium and calcium wasting, nephrocalcinosis, and hypocalcemia [18]. FHHNC follows an autosomal recessive inheritance pattern [19].

In this report, we describe a three-generation family in which nephrocalcinosis emerged as a primary renal manifestation of MEN1. In addition, all three affected individuals harbored an extremely rare *CLDN16* variant, which is now linked to hypomagnesemia in this cohort. These findings highlight the importance of wide genetic screening in patients with complex phenotypes, emphasizing the potential role of co-occurring mutations in modifying disease expression.

## 2. Methods

Relevant clinical and laboratory data were extracted from electronic patient records. Genetic analysis included segregation analysis via Sanger sequencing, Multiplex Ligation-Dependent Probe Amplification (MLPA), and whole-exome sequencing (WES). Genomic DNA was extracted from whole blood, and exon regions were selectively amplified using standard library preparation kits. Whole-exome sequencing was conducted on an Illumina sequencing platform (Illumina, Inc., San Diego (CA), USA) with 100 bp paired-end reads, and sequences were mapped against the UCSC hg38 reference genome. Variants were identified using GATK Haplotype Caller and Germline CNV Caller software (v. 4.5.0.0), allowing for the detection of single-nucleotide variations (SNVs), small insertions and deletions (indels) within ±20 base pairs of coding sequences, and copy-number variants (CNVs) in targeted genes.

The average sequencing depth exceeded 80X, covering >99.8% of the targeted exome. Reportable sequence variants in the proband were validated using di-deoxy DNA sequencing, and variant pathogenicity was assessed using Ingenuity Clinical Insights software (v. 23.0.1, Qiagen Inc., Aarhus, Denmark, EU), the Human Gene Mutation Database (HGMD), and ClinVar (NCBI), (Bethesda (MD), USA). Genomic and amino acid sequences were retrieved from the Ensembl database (EMBL, EU) to further evaluate the potential impact of the detected mutations.

To assess the structural consequences of the mutations, molecular modeling of menin and claudin-16 was performed using PyMOL (v. 2.5.2, Schrödinger LLC, New York, NY, USA) with the PyMOD plugin (v. 3.0.2). Structural predictions were refined using AlphaFold (Technologies Limited, Alphabet Inc., London, UK) to compare sequence alignment differences between the wildtype and mutant proteins [20]. SpliceAI and Pangolin splicing prediction models (https://www.broadinstitute.org, Illumina Inc., San Diego, CA, USA), and MaxEntScan (MIT, Cambridge, MA, USA) were used for in silico detection of possible splicing effect of the CLAUDIN-16 variant [21,22,23].

## 3. Case Series Presentation

The index patient, a 28-year-old female, exhibited a horseshoe kidney and was diagnosed with primary hyperparathyroidism (PTH range: 129–332 pg/mL), accompanied by hypercalcemia (corrected Ca^2+^ 11 mg/dL), hypomagnesemia (Mg^2+^ 1.4 mg/dL), overt magnesiuria (all values > 110 mg/day), hypercalciuria (250–610 mg/day), and nephrocalcinosis. She was initially treated with hydrochlorothiazide (HCTZ) for hypercalciuria; however, this led to worsening of hypomagnesemia, magnesiuria, and hypercalcemia. Consequently, HCTZ was discontinued, and cinacalcet was initiated at a dose of 60 mg per day. In 2021, a hormonal evaluation revealed normal levels of prolactin, IGF-1, gastrin, calcitonin, growth hormone, insulin, and metanephrines.

Her 48-year-old mother also presented with primary hyperparathyroidism (PTH range: 100–444 pg/mL), hypercalcemia (corrected Ca^2+^: 11.7 mg/dL), hypomagnesemia (Mg^2+^: 1.5 mg/dL), magnesiuria (all values > 126 mg/day), and nephrocalcinosis. She underwent parathyroid adenoma resection at the age of 35, which led to a partial improvement in hyperparathyroidism; however, hypercalcemia, hypercalciuria, and magnesiuria persisted, necessitating the initiation of cinacalcet. In 2021, genetic testing identified a *Menin* mutation as discussed below. This finding prompted a comprehensive re-evaluation, which revealed a pancreatic neuroendocrine tumor, elevated serum gastrin and chromogranin A levels, and hyperprolactinemia due to a pituitary adenoma. The index patient’s 84-year-old grandmother underwent parathyroid adenoma resection at the age of 55 due to hypercalcemia. Despite surgery, she experienced persistent hyperparathyroidism (PTH range: 90–303 pg/mL), which gradually led to nephrocalcinosis. She also developed diabetes mellitus and eventually required dialysis at the age of 77. A low serum magnesium level (Mg^2+^ 1.5 mg/dL) and magnesiuria were also observed up until she started dialysis.

All three patients exhibited a 12-base deletion spanning the intron 9 and exon 10 boundaries of the *MEN1* gene (c.1351-3_1359delCAGGTGCGGCAG), which was identified with MPLA. This variant (referenced as rs2136094253) has been reported in the literature in at least one family affected with MEN1 [24]. It was also reported in ClinVar (Variation ID: 1066873) and is absent from the Genome Aggregation Database (gnomAD), indicating that it is not a common polymorphism. This variant deletes the canonical splice acceptor site of intron 9 and part of exon 10 and is considered to be pathogenic/likely pathogenic. Additionally, similar deletions of this splice site are reported in individuals and families affected with MEN1 [24,25,26].

Another variant regarding the *CLDN16* gene (c.114+13C>G, rs369250510) was also detected in all three patients. This is an intronic variant, with a frequency of 0.000188, absent in gnomAD genomes, that co-segregated with renal hypomagnesemia in all affected family members, suggesting a possible modifying role in disease presentation. The variant c.114+13C>G is located 13 bp downstream of exon 1 in CLDN-16 (NM_001378493.1). It is near the splice donor site, in an area that might influence splicing regulatory elements. MaxEntScan scores were very low (Supplementary File S2). A more positive score, for example, above 4, would be more likely for a splice donor site. After expanding the analysis using SpliceAI, the following outcomes were evident: no predicted weakening of the natural donor site (Donor Loss = 0), a new donor site located about 220 bp upstream (Donor Gain = 0.02), and no effect on acceptor sites (Acceptor Loss/Gain = 0). This was further combined with low conservation (PhyloP = −0.381), low deleteriousness (CADD = 0.979), and lack of a predicted protein effect. Therefore, the CLDN16:c.114+13C>G variant is currently classified as likely benign with respect to splicing function.

**Figure 1:**
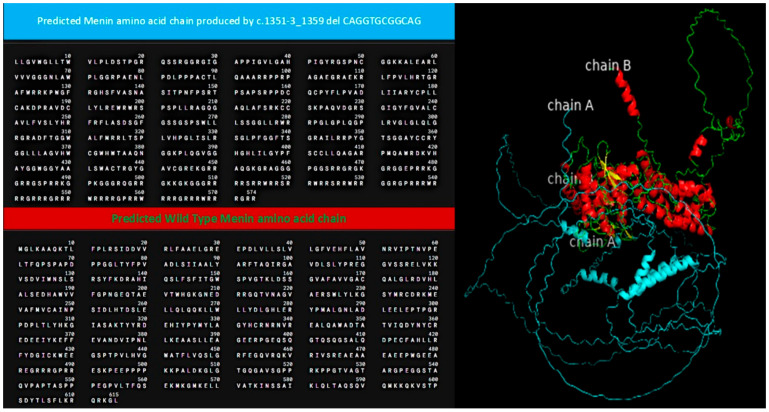
Alignment of predicted menin amino acid sequence (wildtype: blue; mutant: green and red). The genetic alteration produced a predicted sequence lacking an a-helix (chain B).

**Figure 2:**
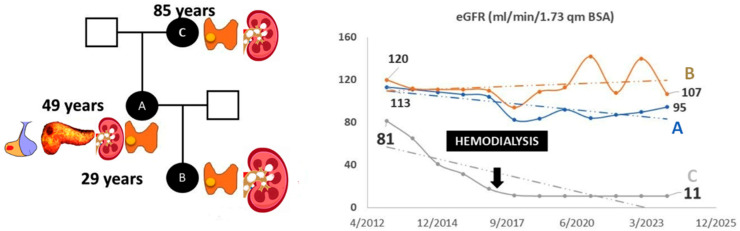
(**Left panel**): clinical information of family members A, B, and C. Affected organs are presented next to the affected individuals as follows: Patient B: parathyroid gland adenoma and nephrolithiasis; Patient A: parathyroid gland adenoma, nephrolithiasis, nephrocalcinosis, pancreatic tumor, and pituitary tumor; Patient C: parathyroid gland adenoma, nephrolithiasis, and nephrocalcinosis. (**Right panel**): renal function over time.

Structural modeling and sequence alignment of the mutated menin protein revealed significant structural alterations. Figure 1 illustrates the altered menin sequence, which lacks an α-helix (chain B), a structural modification that may contribute to its loss of tumor suppressor function. Figure 2 provides an overview of clinical features and renal function progression in the affected family members.

Despite sharing a common genetic basis, renal function in these individuals exhibited distinct and individualized patterns of disease progression, affecting renal function, parathyroid hormone levels, and other clinical manifestations associated with MEN1 syndrome. Notably, all patients presented with hypomagnesemia–magnesiuria, which is not a classic or expected feature of MEN1.

## 4. Discussion

This study highlights the genetic and phenotypic complexity of MEN1 syndrome, particularly in the context of co-occurring gene alterations that may modify disease expression. Genetic analysis identified a 12-base deletion in MEN1, classified as pathogenic/likely pathogenic, in all three affected family members. Additionally, a *CLDN16* variant was found to co-segregate with renal hypomagnesemia in all three patients. Hypomagnesemia is not typically associated with MEN1. Conversely, all three individuals exhibited hypercalcemia, a characteristic feature of MEN1 that contrasts with the typical presentation of hypocalcemia–hypomagnesemia that is seen in FHHNC.

Hypercalcemia in MEN1 may potentially influence renal magnesium handling by activating the calcium-sensing receptor (CaSR) in the renal tubules, leading to renal magnesium wasting as has been seen in a minority of patients with hyperparathyroidism [27]. In the present family, the co-occurrence of a CLDN16 variant may amplify this effect, rendering hypomagnesemia a prominent clinical manifestation. While the CLDN16 variant is likely benign in isolation, it may potentiate magnesiuria in the hypercalcemic milieu of MEN1. Moreover, it could synergistically enhance the hypercalciuric tendencies associated with MEN1, suggesting a potential gene–gene interaction that modulates the renal phenotype observed in these individuals.

### 4.1. Genetic and Clinical Implications

The genetic findings had direct clinical implications. Identification of the MEN1 mutation prompted comprehensive screenings of affected family members, leading to the early diagnosis of a pancreatic neuroendocrine tumor in individual A and its exclusion in the remaining members.

Germline mutations in *MEN1* include frameshift, nonsense, splice-site, and missense variants, accounting for more than 1000 pathogenic mutations [28,29]. A small proportion of MEN1 mutations are sporadic [30]. Menin, the product of the *MEN1* gene, enhances transcription of the cyclin-dependent kinase inhibitors. Furthermore, it interacts with mixed-lineage leukemia (MLL) family proteins. Loss of function of either MLL or menin results in the downregulation of downstream cyclin-dependent kinase inhibitors and deregulated cell growth [31]. Missense mutations of *MEN1* activate the ubiquitin–proteasome pathway through Hsp70 and result in menin degradation [32]. The pluripotent effects on *MEN1* tumor suppression are depicted by the reduced survival of patients having *MEN1* gene mutations when compared with patients having only the clinical phenotype of MEN1 syndrome but no mutation in the *MEN1* gene locus [33]. The majority of *MEN1* mutations are inactivating [34]. Emerging evidence suggests a correlation between phenotypic variability and genomic alterations in *MEN1* [33,34,35]. A proportion of mutations in the *MEN1* gene is located in exon 9 [26,36]. Therefore, exon 9 was considered a site of pathogenic variation in MEN1 syndrome. However, accumulating research revealed that mutations occur throughout the genomic area of *MEN1* [34,37]. Bartsch et al. report that patients with truncating mutations in the amino or carboxyterminal *MEN1* regions exhibit a higher rate of malignant tumors when compared with patients bearing other genetic alterations [38]. The c.1351-1359del mutation in the *MEN1* gene is a deletion that spans from three nucleotides upstream (position c.1351-3) of the exon start to nucleotide c.1359. This alteration within the splice acceptor site of intron 9 leads to improper mRNA splicing. This deletion is predicted to interfere with the proper assembly of the spliceosome. Mutant MEN1 mRNA may undergo degradation due to activation of the nonsense-mediated mRNA decay pathway [39]. This could be a counterbalance mechanism of “controlling” the lack of the *MEN1* gene while increasing the functioning *MEN1* gene expression [40]. Furthermore, there seems to be a small microRNA (miR-24-1) translational “negative feedback loop” intervening with MEN1 mRNA expression [40]. Increased miR-24-1 could “inhibit” MEN1 mRNA expression following the effects of hormones and/or environmental factors, constituting an alternative tumorigenic mechanism, other than the loss of heterozygosity [40]. Mutated menin is truncated and malfunctions. Such truncations lead to structural alterations that alter or even inhibit interaction with transcriptional co-factors such as JunD, MLL1/2, and β-catenin. Loss of these interactions leads to loss of gene expression control and promotes tumorigenesis [4]. In fact, patients with mutations affecting the JunD interaction of menin had a higher risk of dying from neuroendocrine tumors [41]. Menin loss has an epigenetic effect in multiple steps of DNA expression. Specifically, it increases histone acetylation, induces varying effects in multiple histone deacetylase gene expression, increases alternative lengthening of telomeres, and increases DNA methylation [42]. However, most studies focus on the oncogenic potential of specific genetic mutations, with limited data on renal phenotypes associated with MEN1 variants [43]. There is clinical variability and diverse penetrance concerning MEN1 mutations. This fact suggests that there are undetermined factors affecting clinical phenotype [44].

The present study contributes to the growing understanding of MEN1’s genetic complexity, presenting a family in which all three affected members have renal magnesium wasting and carry a novel *CLDN16* mutation in addition to MEN1. The presence of this variant appears to modify the renal phenotype, leading to severe hypercalciuria, early nephrocalcinosis, and hypomagnesemia alongside classical MEN1-associated endocrinopathies [16].

### 4.2. Role of Claudin-16 and the CLDN16 Variant

Claudin-16 is a tight junction protein essential for paracellular calcium and magnesium reabsorption in the thick ascending limb of the loop of Henle [17,18]. Given that hypomagnesemia was observed in all affected family members, the CLDN16 intronic variant (c.114+13C>G) may contribute to renal dysfunction by potentiating hypercalciuria and hypermagnesiuria driven by coexisting hyperparathyroidism.

Although this variant was previously considered benign or of uncertain significance, its association with hypomagnesemia, magnesiuria, and nephrocalcinosis suggests that—under certain genetic or environmental conditions—even seemingly benign variants can influence disease expression [18].

According to ClinVar, the clinical significance of the c.114+13C>G variant has conflicting interpretations. One submission classifies it as of uncertain significance, while another considers it likely benign. This intronic mutation lies 13 nucleotides downstream of exon 1, in close proximity to the canonical splice donor site. Proximal intronic mutations have been shown to disrupt consensus splice sequences and weaken donor/acceptor strength [45]. Multiple predictive tools, however, suggest no significant disruption of canonical splice donor sites, no cryptic splice sites, and a lack of evolutionary conservation. However, due to the co-segregation in all family members with hypomagnesemia, under the context of MEN1 syndrome, this CLDN16 variant may exacerbate or unmask hypomagnesemia. Studies in experimental models of hypercalcemia indicate that claudin-14 is detectable in the thick ascending limb of the loop of Henle (TAL) only in the experimentally induced hypercalcemic state [46]. Interestingly, PTH-induced hypercalcemia induced claudin-14 expression [46]. Additional experimental systems indicate that claudin-14 interacts with claudin-16 but not with claudin-19 in TAL [47]. Claudin-14 abolished the cation selectivity of the claudin-16/19 complex by repressing claudin-16 selectivity [47]. Therefore, in a given experimental design, hyperparathyroidism and hypercalcemia may reduce claudin-16 complex activity and therefore enhance luminal magnesium secretion.

These findings highlight the need for comprehensive genetic evaluation, especially in the presence of concurrent genetic alterations that may modulate disease phenotypes and clinical outcomes.

### 4.3. Clinical Significance and Management Considerations

There are multiple mechanisms that contribute to kidney injury in MEN1 syndrome. The prolonged renal exposure to hypercalcemia leads to nephrocalcinosis, nephrolithiasis, and eventually to hypercalcemic nephropathy [48,49]. In cases of severe hypercalcemia, reduced renal plasma flow due to afferent arteriole vasoconstriction could cause a reduction in glomerular filtration and acute kidney injury [50]. In the presence of primary hyperparathyroidism, there is an increased CKD prevalence in MEN1 [43]. Eller-Vainicher et al. compared renal function in primary hyperparathyroidism (PHPT) patients with that of MEN1-related PHPT patients. MEN1-related PHPT is associated with similar kidney involvement, despite its milder hypercalcemia compared to PHPT without MEN1 [51]. Additionally, there seems to be a benefit in CKD progression after parathyroidectomy in MEN1 patients [43,48]. Therefore, in the presence of nephrocalcinosis or nephrolithiasis in MEN1 syndrome with PHPT, parathyroidectomy is a proposed therapeutic strategy. In fact, patients with prolonged hyperparathyroidism and MEN1 have an increased risk for early nephrolithiasis and more severe renal complications compared to hyperparathyroidism cases without MEN1 syndrome [52]. Five percent of MEN1 patients aged 20–39 have CKD stage 3. For the same age group in the general US population, the prevalence of CKD stage 3 is about 0.4%. In ages 40–59, 10% of MEN1 patients had CKD stage 3 or worse, while for the same age group in the general population, the prevalence was about 2.3% [43]. These findings indicate the earlier and higher propensity towards CKD progression in MEN1 patients with hypercalcemia. On the other hand, in the absence of PHPT, CKD progression can occur in the context of other CKD risk factors, even in the absence of extended nephrolithiasis [53]. MEN1 expression has been linked with the development of renal fibrosis in experimental mouse models of unilateral ureteral obstruction (UUO), induced tubulointerstitial fibrosis (TIF), and diabetes [54,55]. Menin, MLL1, and H3K4me3 proteins were reduced in the kidneys of UUO and diabetic mice [54]. In addition, MEN1 deletion caused increased epithelial-to-mesenchymal transition, which was dependent on chromatin modification mediated through H3K4me3. Menin exerts pluripotent actions in mouse models with interstitial fibrosis. Specifically, menin’s absence induces a G2/M cell cycle arrest in renal tubular epithelial cells, activates the Janus Kinase signaling pathway, and increases hepatocyte growth factor-dependent Adamts5 expression. Interestingly, the administration of hepatocyte growth factor restored tissue Adamts5 in this experimental model and ameliorated tubulointerstitial fibrosis [54]. In another experimental study of tubulointerstitial fibrosis involving UUO mice, disruption of the MLL1–menin interaction resulted in attenuated renal tubulointerstitial fibrosis through TGF-β1 and E-cadherin signaling [55]. The presence of one functioning allele in MEN1 patients could suggest an additional susceptibility to the development of renal fibrosis. Concerning patients with end-stage kidney disease and MEN1 syndrome, a definite diagnosis poses a clinical challenge. ESKD patients can also have secondary HPT with multiglandular involvement. Historically, the administration of active vitamin D supplements can cause severe and life-threatening hypercalcemia in MEN1 patients with ESKD. However, the presence of endocrine tumors and a positive family history should raise clinical suspicion.

Several limitations must be acknowledged. First, the small sample size—restricted to three related individuals—limits the generalizability of the findings. To validate these observations and better understand potential genotype–phenotype correlations between MEN1 and renal involvement, larger cohort studies involving MEN1 patients with concurrent *CLDN16* alterations are warranted. While such dual-genotype cases may be rare, the increasing adoption of whole-exome sequencing (WES) may facilitate the identification of similar genetic constellations in broader populations.

Second, the mechanistic contribution of the *CLDN16* variant to nephrocalcinosis remains speculative. Functional studies examining the impact of the c.114+13C>G variant on claudin-16 expression, localization, and activity in renal tissue could provide critical insights into whether this variant acts as a modifier of the MEN1-associated renal phenotype [17,18].

## 5. Conclusions

This case series highlights the genetic and phenotypic complexity of MEN1 syndrome, particularly when compounded by additional genetic variants such as CLDN16. The novel intronic variant c.324+13C>G, identified in all three affected family members, may serve as a modifier of the MEN1 phenotype, with specific impact on renal involvement and magnesium handling.

The pleiotropic effects of MEN1 mutations—when combined with alterations in genes regulating renal ion transport—highlight the importance of comprehensive genetic screening and multidisciplinary management in affected families. These findings also emphasize the value of integrating nephrological assessment into the routine evaluation of MEN1 patients.

Future research should focus on elucidating the molecular mechanisms underlying these complex interactions, with the ultimate goal of developing more targeted and personalized therapeutic interventions for MEN1 patients with co-occurring genetic alterations.

## Data Availability

The data that support the findings of this study are available from the corresponding author upon reasonable request.

## Supplementary Materials

The following supporting information can be downloaded at: https://www.mdpi.com/article/10.3390/jcm14155447/s1: File S1: SpliceAI & Pangolin results, File S2: MaxEntScan results for in silico detection of possible splicing effect of the CLAUDIN-16 variant.
