# Supplementary material for: Familial MEN1 Syndrome with Atypical Renal Features and a Coexisting CLDN16 Variant: A Case Series
Source: J Clin Med. 2025 Aug 2;14(15):5447. doi: 10.3390/jcm14155447 (PMC12347269; doi:10.3390/jcm14155447)
Supplement: Supplementary file 1 [file jcm-14-05447-s001.zip › File S1. SpliceAI Lookup CLAUDIN16.pdf]

Enter a variant below to see its SpliceAI <https://pubmed.ncbi.nlm.nih.gov/30661751>, Pangolin <https://github.com/tkzeng/Pangolin>, and other scores
[more details] <#>

SpliceAI <https://github.com/illumina/SpliceAI> is a copyrighted work by Illumina and is provided under GPLv3 and CC BY NC 4.0 licenses for academic and non-commercial use. This web service is maintained by the TGG <https://the-tgg.org/> at the Broad Institute <https://www.broadinstitute.org/>. It runs the SpliceAI and Pangolin splicing prediction models on user-specified variants and then displays the scores.

For more information about SpliceAI, see Jaganathan et al, Cell 2019 <https://doi.org/10.1016/j.cell.2018.12.015> and this recorded talk <https://www.youtube.com/watch?v=oJvhj-tYbBI> by Kishore Jaganathan.
For more information about Pangolin, see Zeng & Li 2022 <https://genomebiology.biomedcentral.com/articles/10.1186/s13059-022-02664-4>.

Under the hood, this service runs modified versions of SpliceAI and Pangolin which have added support for multi-nucleotide variants (MNVs), provide access to raw scores in addition to delta scores, and have other customizations:
https://github.com/bw2/SpliceAI <https://github.com/bw2/SpliceAI>
https://github.com/bw2/Pangolin <https://github.com/bw2/Pangolin>

The visualizations of SpliceAI and Pangolin results are implemented as custom IGV.js <https://github.com/igvteam/igv.js> tracks at:
https://github.com/bw2/igv.js <https://github.com/bw2/igv.js/pull/1>

We no longer use the Illumina precomputed SpliceAI score tables since they are based on an old version of Gencode.

NOTE: This service supports no more than a handful of queries per-user per-minute. If you need to batch-process many variants, please use the source code <https://github.com/broadinstitute/SpliceAI-lookup/blob/master/README.md#local-install> to set up your own local instance of the API server or just run the SpliceAI and Pangolin models directly.

Examples (on hg38):

chr8-140300616-T-G

6 31740453 G T

NM_001089.3(ABCA3):c.875A>T (p.Glu292Val) [show more examples] <#>

8:140300616 T>G 'chr' /prefix is optional/

1:930130:C:G /position with overlapping genes/

1-1042601-A-AGAGAG /insertion of GAGAG/

1-1042466-GGGC-G /deletion of GGC/

NM_000249.4(MLH1):c.116G>A /another HGVS example/

Genome version:

hg19

hg38

Gencode:

basic

comprehensive

//

Max distance:

//

masked scores

//

REF & ALT scores

//

Submit

SpliceAI-lookup/issues <https://github.com/broadinstitute/SpliceAI-lookup/issues>: issues or feature requests for this website

December 5, 2024

- add PrimateAI-3D <https://www.ncbi.nlm.nih.gov/pmc/articles/PMC10187174/>, PromoterAI, AlphaMissense <https://www.science.org/doi/10.1126/science.adg7492> and other scores
- provide a table with detailed SpliceAI scores for insertion variants whenever donor or acceptor gain is predicted to occurs within the inserted sequence itself. This addresses issue #84 <https://github.com/broadinstitute/SpliceAI-lookup/issues/84>.

MAJOR BUG FIX: Between April and November of 2024, *Pangolin* scores were incorrectly computed for ~28% of hg38 variants and ~5% of hg19

variants due to a bug related to multiple requests being processed in

parallel. This issue has now been fixed.

SpliceAI scores were fine and were not affected by this bug.

[show older updates] <#> *November 3, 2024*

- added choice of Gencode basic or comprehensive <https://genome.ucsc.edu/FAQ/FAQgenes.html#gencode> transcripts
- updated to Gencode v47

June 20, 2024

- fixed SpliceAI visualizations to clarify positions of predicted splicing changes. See issue #70 <https://github.com/broadinstitute/SpliceAI-lookup/issues/70> for details.

June 3, 2024

- show the input variant's VEP <https://useast.ensembl.org/info/docs/tools/vep/index.html> consequence in the results table
- the Gencode gene track is now optional in the visualizations
- updated everything to Gencode v46 <https://www.gencodegenes.org/human/releases.html>

March 7, 2024

- added warning for insertion variants with delta scores ≥ 0.2 and position = 0bp saying that they may be difficult to interpret due to issue #67 <https://github.com/broadinstitute/SpliceAI-lookup/issues/69>. Thanks to @SophieCandille for the issue report and example variant: 2:47790924 C>CAGTTG <https://spliceailookup.broadinstitute.org/

#variant=NM_000179.3%3Ac.261-3_261-2insAGTTG&hg=38&distance=500&mask=0&ra=1>

- moved server to Google Cloud Run to better support higher usage

November 30, 2023

- changed default search settings to "not masked" due to confusion <https://github.com/broadinstitute/SpliceAI-lookup/issues/59#issuecomment-1815667021> and issues <https://github.com/Illumina/SpliceAI/issues/27#issuecomment-547089167> with how masked scores are computed.
- when looking at hg19 variants, the visualization checkboxes now make it clear which IGV.js tracks are or aren't available on hg19.

October 5, 2023

- fixed Pangolin bug identified by @kazmiekr <https://github.com/invitae/pangolin/pull/5> where variants that overlap more than one gene showed incorrect masked scores
- @pj-sullivan <https://github.com/pj-sullivan> added controls to show/hide transcripts
- added MANE Select transcript labels
- updated to gencode v44 "basic" annotations (instead of comprehensive). These have fewer transcripts per gene.
- added igv.js visualizations

May 22, 2023

- changed defaults to 'masked' and max distance to 500bp
- retired *Illumina precomputed scores* since they were created using Gencode v24 and max distance = 50bp and so can differ from computed

scores which are based on the latest Gencode.

May 12, 2023

- added REF & ALT score columns to show SpliceAI's underlying predictions for each haplotype (using SpliceAI PR by @h-joshi <https://github.com/Illumina/SpliceAI/pull/92>). The Δ score column is the difference between these two scores.
- added support for MNPs (see issue #1 <https://github.com/broadinstitute/SpliceAI-lookup/issues/1> & SpliceAI PR by @kdahlo <https://github.com/Illumina/SpliceAI/pull/119>)
- updated to Gencode v43 <https://www.gencodegenes.org/human/releases.html> (was previously on v42)

May 10, 2023

- fixed bug where "raw" Pangolin scores were shown regardless of whether the user selected "raw" or "masked".

Feb 5, 2023

- show Pangolin scores <https://github.com/tkzeng/Pangolin> in addition to SpliceAI scores.

June 2, 2021

- show RefSeq ids for the subset of Ensembl ENST ids (~30%) that have one or more matching RefSeq NM ids according to Ensembl. See issue #8 <https://github.com/broadinstitute/SpliceAI-lookup/issues/8> for details.

April 11, 2021

- show gray background for non-coding transcripts

- switch to showing all non-coding transcripts. (Previously, transcripts with Gencode biotypes like /lncRNA/, /processed_pseudogene/, /processed_transcript/, /retained_intron/, and /nonsense_mediated_decay/ were filtered out if they overlapped /protein_coding/ transcripts). See issue #6 <https://github.com/broadinstitute/SpliceAI-lookup/issues/6#issuecomment-816942824> for details.

[hide older updates] <#>

Related web tools:
liftover <https://liftover.broadinstitute.org/>: for variants/positions/intervals (hg19 <=> hg38 <=> T2T)
TGG Viewer <https://tgg-viewer.broadinstitute.org/>: igv.js-based web viewer for public reference tracks and private data in Google Storage buckets. Has custom track types for RNA-seq splice junctions <https://github.com/igvteam/igv.js/pull/1019#issue-521944897> and gCNV <https://github.com/igvteam/igv.js/pull/1055#issue-560042328> variants.
SpliceAI scores: //
Variant Gene
= MANE Select transcript = non-coding transcript
Δ type Δ score // position // REF score // ALT score //
NM_001378493.1(CLDN16):c.114+13C>G

/⇒ 3:190388456 C>G/

non coding transcript exon variant <https://www.ensembl.org/info/genome/variation/prediction/predicted_data.html>

UCSC <https://genome.ucsc.edu/cgi-bin/hgTracks?db=hg38&position=chr3:190388456>, gnomAD <https://gnomad.broadinstitute.org/variant/3-190388456-C-G?dataset=gnomad_r4>

CLDN16 (ENSG00000113946.4 <https://useast.ensembl.org/Homo_sapiens/Gene/Summary?g=ENSG00000113946> / ENST00000264734.3 <https://useast.ensembl.org/Homo_sapiens/Transcript/Summary?t=ENST00000264734> / NM_006580.4 <https://www.ncbi.nlm.nih.gov/search/all/?term=NM_006580.4>)

protein coding <https://www.gencodegenes.org/pages/biotypes.html>
MANE Select transcript <https://www.ncbi.nlm.nih.gov/refseq/MANE>
(plus strand)

OMIM <https://www.omim.org/search?search=CLDN16>, GTEx <https://gtexportal.org/home/gene/CLDN16>, gnomAD <https://gnomad.broadinstitute.org/gene/CLDN16?dataset=gnomad_r4>, ClinGen <https://search.clinicalgenome.org/kb/genes?page=1&size=25&search=CLDN16>, Ensembl <https://useast.ensembl.org/Homo_sapiens/Gene/Summary?g=CLDN16>, Decipher <https://www.deciphergenomics.org/gene/CLDN16>, GeneCards <https://www.genecards.org/cgi-bin/carddisp.pl?gene=CLDN16>

Acceptor Loss	0.00	
Donor Loss	0.00	-13 bp
Acceptor Gain	0.00	-219 bp
Donor Gain	0.02	-220 bp

NM_001378493.1(CLDN16):c.114+13C>G

/⇒ 3:190388456 C>G/

non coding transcript exon variant <https://www.ensembl.org/info/genome/variation/prediction/predicted_data.html>

UCSC <https://genome.ucsc.edu/cgi-bin/hgTracks?db=hg38&position=chr3:190388456>, gnomAD <https://gnomad.broadinstitute.org/variant/3-190388456-C-G?dataset=gnomad_r4>

CLDN16 (ENSG00000113946.4 <https://useast.ensembl.org/Homo_sapiens/Gene/Summary?g=ENSG00000113946> / ENST00000456423.2 <https://useast.ensembl.org/Homo_sapiens/Transcript/Summary?t=ENST00000456423>)

protein coding <https://www.gencodegenes.org/pages/biotypes.html>
(plus strand)

OMIM <https://www.omim.org/search?search=CLDN16>, GTEx <https://gtexportal.org/home/gene/CLDN16>, gnomAD <https://gnomad.broadinstitute.org/gene/CLDN16?dataset=gnomad_r4>, ClinGen <https://search.clinicalgenome.org/kb/genes?page=1&size=25&search=CLDN16>, Ensembl <https://useast.ensembl.org/Homo_sapiens/Gene/Summary?g=CLDN16>, Decipher <https://www.deciphergenomics.org/gene/CLDN16>, GeneCards <https://

www.genecards.org/cgi-bin/carddisp.pl?gene=CLDN16> Acceptor Loss 0.00

Donor Loss 0.00 -13 bp

Acceptor Gain 0.00 -219 bp

Donor Gain 0.02 -220 bp

NM_001378493.1(CLDN16):c.114+13C>G

/⇒ 3:190388456 C>G/

non coding transcript exon variant <https://www.ensembl.org/info/genome/variation/prediction/predicted_data.html>

UCSC <https://genome.ucsc.edu/cgi-bin/hgTracks?db=hg38&position=chr3:190388456>, gnomAD <https://gnomad.broadinstitute.org/variant/3-190388456-C-G?dataset=gnomad_r4>

CLDN16 (ENSG00000113946.4 <https://useast.ensembl.org/Homo_sapiens/Gene/Summary?g=ENSG00000113946> / ENST00000468220.1 <https://useast.ensembl.org/Homo_sapiens/Transcript/Summary?t=ENST00000468220>)

protein coding CDS not defined <https://www.gencodegenes.org/pages/biotypes.html>
(plus strand)

OMIM <https://www.omim.org/search?search=CLDN16>, GTEx <https://gtexportal.org/home/gene/CLDN16>, gnomAD <https://

gnomad.broadinstitute.org/gene/CLDN16?dataset=gnomad_r4>, ClinGen <https://search.clinicalgenome.org/kb/genes?page=1&size=25&search=CLDN16>, Ensembl <https://useast.ensembl.org/Homo_sapiens/Gene/Summary?g=CLDN16>, Decipher <https://www.deciphergenomics.org/gene/CLDN16>, GeneCards <https://www.genecards.org/cgi-bin/carddisp.pl?gene=CLDN16> Acceptor Loss 0.06 -219 bp

Donor Loss 0.06 -13 bp

Acceptor Gain 0.00

Donor Gain 0.00

NM_001378493.1(CLDN16):c.114+13C>G

/⇒ 3:190388456 C>G/

non coding transcript exon variant <https://www.ensembl.org/info/genome/variation/prediction/predicted_data.html>

UCSC <https://genome.ucsc.edu/cgi-bin/hgTracks?db=hg38&position=chr3:190388456>, gnomAD <https://gnomad.broadinstitute.org/variant/3-190388456-C-G?dataset=gnomad_r4> ENSG00000297357 (ENSG00000297357.1 <https://useast.ensembl.org/Homo_sapiens/Gene/Summary?g=ENSG00000297357> / ENST00000747317.1 <https://useast.ensembl.org/Homo_sapiens/Transcript/Summary?t=ENST00000747317>)

lncRNA <https://www.gencodegenes.org/pages/biotypes.html>

(minus strand)

OMIM <https://www.omim.org/search?search=ENSG00000297357>, GTEx <https://gtexportal.org/home/gene/ENSG00000297357>, gnomAD <https://gnomad.broadinstitute.org/gene/ENSG00000297357?dataset=gnomad_r4>, ClinGen <https://search.clinicalgenome.org/kb/genes?page=1&size=25&search=ENSG00000297357>, Ensembl <https://useast.ensembl.org/Homo_sapiens/Gene/Summary?g=ENSG00000297357>, Decipher <https://www.deciphergenomics.org/gene/ENSG00000297357>, GeneCards <https://www.genecards.org/cgi-bin/carddisp.pl?gene=ENSG00000297357> Acceptor Loss 0.00 -271 bp

Donor Loss 0.00 -55 bp

Acceptor Gain 0.00 -19 bp

Donor Gain 0.00 -215 bp

MANE Select Transcript

All Transcripts

Pangolin scores: //

Variant Gene Δ type Δ score // position // REF score // ALT score //

NM_001378493.1(CLDN16):c.114+13C>G

/⇒ 3:190388456 C>G/

non coding transcript exon variant <https://www.ensembl.org/info/genome/variation/prediction/predicted_data.html>

UCSC <https://genome.ucsc.edu/cgi-bin/hgTracks?db=hg38&position=chr3:190388456>, gnomAD <https://gnomad.broadinstitute.org/variant/3-190388456-C-G?dataset=gnomad_r4>
CLDN16 (ENSG00000113946.4 <https://useast.ensembl.org/Homo_sapiens/Gene/Summary?g=ENSG00000113946> / ENST00000264734.3 <https://useast.ensembl.org/Homo_sapiens/Transcript/Summary?t=ENST00000264734> / NM_006580.4 <https://www.ncbi.nlm.nih.gov/search/all/?term=NM_006580.4>)

protein coding <https://www.gencodegenes.org/pages/biotypes.html>
MANE Select transcript <https://www.ncbi.nlm.nih.gov/refseq/MANE>
(plus strand)

OMIM <https://www.omim.org/search?search=CLDN16>, GTEx <https://gtexportal.org/home/gene/CLDN16>, gnomAD <https://gnomad.broadinstitute.org/gene/CLDN16?dataset=gnomad_r4>, ClinGen <https://search.clinicalgenome.org/kb/genes?page=1&size=25&search=CLDN16>, Ensembl <https://useast.ensembl.org/Homo_sapiens/Gene/Summary?g=CLDN16>, Decipher <https://www.deciphergenomics.org/gene/CLDN16>, GeneCards <https://www.genecards.org/cgi-bin/carddisp.pl?gene=CLDN16> Splice Loss 0.03
-13 bp

Splice Gain 0.00 -5 bp

NM_001378493.1(CLDN16):c.114+13C>G

/⇒ 3:190388456 C>G/

non coding transcript exon variant <https://www.ensembl.org/info/genome/variation/prediction/predicted_data.html>

UCSC <https://genome.ucsc.edu/cgi-bin/hgTracks?db=hg38&position=chr3:190388456>, gnomAD <https://gnomad.broadinstitute.org/variant/3-190388456-C-G?dataset=gnomad_r4>

ENSG00000297357 (ENSG00000297357.1 <https://useast.ensembl.org/Homo_sapiens/Gene/Summary?g=ENSG00000297357> / ENST00000747317.1 <https://useast.ensembl.org/Homo_sapiens/Transcript/Summary?t=ENST00000747317>)

lncRNA <https://www.gencodegenes.org/pages/biotypes.html>
(minus strand)

OMIM <https://www.omim.org/search?search=ENSG00000297357>, GTEx <https://gtexportal.org/home/gene/ENSG00000297357>, gnomAD <https://gnomad.broadinstitute.org/gene/ENSG00000297357?dataset=gnomad_r4>, ClinGen <https://search.clinicalgenome.org/kb/genes?page=1&size=25&search=ENSG00000297357>, Ensembl <https://

useast.ensembl.org/Homo_sapiens/Gene/Summary?g=ENSG00000297357>,
Decipher <https://www.deciphergenomics.org/gene/ENSG00000297357>,
GeneCards <https://www.genecards.org/cgi-bin/carddisp.pl?
gene=ENSG00000297357> Splice Loss 0.01 -271 bp
Splice Gain 0.06 -19 bp

Other scores: //
Variant Method Score Points
NM_001378493.1(CLDN16):c.114+13C>G

/⇒ chr3:190388456 C>G/

CADD 0.979 -2 //
PhyloP -0.381 -2 //
PromoterAI -0.01 //

AlphaMissense, PolyPhen (max), PrimateAI-3D, REVEL, and SIFT (max) scores are not available for this variant //

Visualizations:
SpliceAI & Pangolin results // GTEx RNA: average per sample // GTEx RNA: all splice junctions // SpliceAI: precomputed scores // Reference // variant track

SpliceAI REF & ALT scores

SpliceAI Δ scores

Pangolin Δ scores

blood

fibroblasts

muscle

LCLs

brain cortex

blood

fibroblasts

muscle

LCLs

brain cortex

gain ≥ 0.5

loss ≥ 0.5

gain ≥ 0.2

loss ≥ 0.2

gencode v47 genes

100-mer mappability

segmental duplications

Update

To see *documentation* or *legends* for each track, click on the rectangle that contains the track name.

OK

IGV;

hg38

458 bp

cursor guide

center line

track labels

ROI Table

Sample Info

Sample Names

Save SVG

190,388,297

OK

Zoom in to see features

Refseq

Zoom in to see features

Variant

Zoom in to see features

SpliceAI REF/ALT

Zoom in to see features

SpliceAI Δ

Zoom in to see features

Pangolin Δ

Regions of Interest

Chr

Start

End

Description

Go To

OK

Cancel

Minimum

Maximum

OK

Cancel

This section shows AlphaMissense, PrimateAI-3D, and PromoterAI scores retrieved from publicly-available lookup tables generated by the authors of these models, as well as CADD, PhyloP, PolyPhen, REVEL, and SIFT scores retrieved from the gnomAD and myvariant.info APIs
