# Supplementary material for: Familial MEN1 Syndrome with Atypical Renal Features and a Coexisting CLDN16 Variant: A Case Series
Source: J Clin Med. 2025 Aug 2;14(15):5447. doi: 10.3390/jcm14155447 (PMC12347269; doi:10.3390/jcm14155447)
Supplement: Supplementary file 1 [file jcm-14-05447-s001.zip › File S2. MAXENTCLAUDIN16.pdf]

>NM_001378493.1 Homo sapiens claudin 16 (CLDN16), transcript variant 3, mRNA

GCATTTTAGAGAGCTTTTGTCTGTGAAGGCTAAAAGAATGGCCACTTGTAGAGTATACTTGAAAAAGAAT
MAXENT: -26.35 MDD: -14.53 MM: -13.32 WMM: -14.43

TAGGTTTAGTCTAAATTGTATCATTTGACCTTCAGGATCTCTTTCAGTCCTAGGTGGAAGACAATTTTAA
MAXENT: -1.75 MDD: 2.68 MM: 0.53 WMM: -1.25

GGAAAAAAAACCTACTCTTTCATACCTCACCATTTTCGTTTCCATAAATTGTCCTCCTTTCATCAGCTCA
MAXENT: -20.81 MDD: -13.49 MM: -15.20 WMM: -14.74

TCTCTGAAATTCTACAAGGAGATGATTCCTGCCCTCAAACATCGGACTCCAAGTTCTTGAGTTTTGGGAC
MAXENT: -17.69 MDD: -17.00 MM: -7.56 WMM: -9.88

TCAGACTGGCTTTCTTGCTCCTCAGCTTGCAGATGAACTACTGTGGGATCTTGTGATCATGACTGAACTC
MAXENT: -20.33 MDD: -10.81 MM: -13.97 WMM: -11.06

GTTGTTGGCCACCTGTTTGGAAAACCATTCTCTTGTGGTTGGATCTAACACATACAGCATGTGAAGGTAC
MAXENT: -15.91 MDD: -3.12 MM: -9.60 WMM: -2.84

CAGAAACACAGAAGACTGACACCCGCCACTTAAGTGGGGCCAGGGCTGGTGTCTGCCCATGTTGCCATC
C MAXENT: -9.08 MDD: -3.79 MM: -9.54 WMM: -10.96

TGATGGGCTGCTTGCCACAATGAGGGATCTTCTTCAATACATCGCTTGCTTCTTTGCCTTTTTCTCTGCT
MAXENT: -29.38 MDD: -20.87 MM: -18.32 WMM: -18.15

GGGTTTTTGATTGTGGCCACCTGGACTGACTGTTGGATGGTGAATGCTGATGACTCTCTGGAGGTGAGC
A MAXENT: -16.50 MDD: -8.23 MM: -9.53 WMM: -11.65

CAAAATGCCGAGGCCTCTGGTGGGAATGCGTCACAAATGCTTTTGATGGGATTCGCACCTGTGATGAGT
A MAXENT: -29.89 MDD: -17.79 MM: -20.36 WMM: -18.84

CGATTCCATACTTGCGGAGCATCCCTTGAAGCTGGTGGTAACTCGAGCGTTGATGATTACTGCAGATATT
MAXENT: -24.67 MDD: -10.13 MM: -16.25 WMM: -11.84

CTAGCTGGGTTTGGATTTCTCACCCTGCTCCTTGGTCTTGACTGCGTGAAATTCCTCCCTGATGAGCCGT
MAXENT: -16.78 MDD: -9.78 MM: -11.03 WMM: -8.14

ACATTAAAGTCCGCATCTGCTTTGTTGCTGGAGCCACGTTACTAATAGCAGGTACCCCAGGAATCATTGG
MAXENT: -10.00 MDD: -5.13 MM: -5.17 WMM: -5.70

CTCTGTGTGGTATGCTGTTGATGTGTATGTGGAACGTTCTACTTTGGTTTTGCACAATATATTTCTTGGT
MAXENT: -40.44 MDD: -19.87 MM: -25.15 WMM: -20.13

ATCCAATATAAATTTGGTTGGTCCTGTTGGCTCGGAATGGCTGGGTCTCTGGGTTGCTTTTTGGCTGGAG
MAXENT: -23.13 MDD: -14.18 MM: -13.88 WMM: -15.04

CTGTTCTCACCTGCTGCTTATATCTTTTTAAAGATGTTGGACCTGAGAGAAACTATCCTTATTCCTTGAG
MAXENT: -16.73 MDD: -6.63 MM: -9.43 WMM: -10.11

GAAAGCCTATTCAGCCGCGGGTGTTTCCATGGCCAAGTCATACTCAGCCCCTCGCACAGAGACGGCCAAA
MAXENT: -33.20 MDD: -16.55 MM: -24.45 WMM: -19.88

ATGTATGCTGTAGACACAAGGGTGTAAAATGCACGTTTCAGGGTGTGTTTGCATATGATTTAATCAATCA
MAXENT: -23.18 MDD: -16.31 MM: -17.06 WMM: -17.18

GTATGGTTACATTGATAAAATAGTAAGTCAATCCAGGAACAGTTATTTAGAATTCATATTGAATTAAATT
MAXENT: -34.16 MDD: -20.17 MM: -20.20 WMM: -19.06

AATTGCTAGCTTAATCAAAATGTTTGATTCTCCTATACTTTTTCTTTCTATTACTCTTATATTTTCCCGT
MAXENT: -30.69 MDD: -21.57 MM: -22.52 WMM: -19.41

CATTCTCTCTGCTAACCTTCCACCTTATGCACACACTTTCCCTATATTTTAAGATAAGTCTGCTAGGATG
MAXENT: -35.29 MDD: -22.48 MM: -27.63 WMM: -20.37

TAGAAATATTTGTTTGTGATTTCTATATAGCTATTAGAGATTATGACATAGTAATATTAAAATGAAATGA
MAXENT: -11.48 MDD: -7.99 MM: -11.91 WMM: -11.73

TACTTAAACAGAAAGCAATTTCCAAAGAGGCCAGGGACCCTAATCTTTGAAGAGATGAAGAAACTTACTT
MAXENT: -13.80 MDD: -2.83 MM: -6.81 WMM: -5.00

TTCTCCCTGGCTTTTGGTTCACTTTTTGTACTTTTAACAAGTGGGTGAATTATTTGATAATTTTGAGGAA
MAXENT: -36.76 MDD: -20.88 MM: -22.99 WMM: -22.46

GATTATTCTTTTAAATTCAAACTAGTATGTCAATGCCTACCATTACTCTGATTATATTAAAACAGAAAAA
MAXENT: -30.39 MDD: -25.21 MM: -27.90 WMM: -20.67

GGAAATAACAACTTCGTATACCAGCCACTGGTGAGAGTTAAAGACAAGAGCTGCCCCCCCACCCCCAAAT
MAXENT: -33.30 MDD: -19.69 MM: -22.88 WMM: -19.57

GTCAAAGGCAAATGCTAAATTGATACTGGAGCTCGTGGTGACTTTCTACCTCACTAACAACATAAGGGAT
MAXENT: -16.56 MDD: -10.89 MM: -12.78 WMM: -12.99

CTCCATATTATTTCACCACTATTCTAGCTTTGCTGATATATTGCCAAATGATTAGACTACAGAATAGTTC
MAXENT: -28.42 MDD: -18.28 MM: -24.72 WMM: -17.13

AACCAGAGAATTTACTCATTTATTGATTAAACATCCAAATACTATTGTAATATACTATGTTAAAATTCAT
MAXENT: -11.33 MDD: -6.98 MM: -9.73 WMM: -9.33

CAATTCAAGTGCCCACACACCACTGAATCATCAGCACCAAGCAATATATTAGACATATGGCAAAATTCAA
MAXENT: -17.91 MDD: -5.33 MM: -12.35 WMM: -7.50

CAAATATATTTTGATATAAATAAATAAACGTTCACGACTTTACTTAAAAAATCAATGTTGCGGCTGGGCA
MAXENT: -6.21 MDD: -2.70 MM: -4.62 WMM: -4.67

CGGTAGCTCGCGTCTGTAATCCCCGCACTTTGGGAGGCCAAGGCGGGTGGATCACGAGGTCAAGAGACGG MAXENT: -17.20 MDD: -11.31 MM: -14.26 WMM: -15.39

AGACCATCCTGGCTAACATGGTGAAACCCTGTCTCTACTAAAAATACAAAAATTAGCCGGGCGTGGTGGC MAXENT: -20.14 MDD: -15.15 MM: -14.75 WMM: -16.77

GGTGCCTGTAGTCCCAGCTACTCGGGAGGCTGAGGCAGGAGAATCGTTTGAACCCAGGAGGTGGAGGTTG MAXENT: -14.47 MDD: -8.68 MM: -12.80 WMM: -9.75

CAGTGAGCGGAGATCGCACCATTGCACTCCAGTCTGGCAACAGAGCGAGACTCCATCTCAAAAAACAAAA MAXENT: -8.78 MDD: -4.77 MM: -9.46 WMM: -10.22

ATAAATAAATAAATAAATATTCTTCATAAAATGTGGGTTTTGGGGAAAATATAGAATTACATATACATTT MAXENT: -30.05 MDD: -19.29 MM: -22.71 WMM: -18.61

AACGAAGTCGCTAATGACATTTCATTCATATTCATAATGTAACCATCTTGAATTTTTTTAATTGTAGCGA MAXENT: -11.65 MDD: -2.91 MM: -7.62 WMM: -5.84

TTTTAAAAATGTTTGTAAAATTTAATTTCCAGTTTTCTAATTACTTGTCAGTCACATTAATAACATTAGT MAXENT: -23.30 MDD: -22.01 MM: -16.49 WMM: -16.60

ACCTTTATGGTACCCTTGCAGTACCTGAAAAGAATATCAACCTGAAAAGAATATCAACTCACCCAGAAAT MAXENT: -25.31 MDD: -10.73 MM: -16.24 WMM: -10.79

TAGTTCTTTGAAAAAAAAGAAATTAAGTTGTGAATTTCTAAAGACCTTGAAATAAGTGTTTCAAATTTAA MAXENT: -9.63 MDD: -5.93 MM: -9.15 WMM: -8.84

AGAACAAAGAATGATGTGAAAATGAGATTATGATTCCTACTACATGAATTAACGTTTCGAGATTGCTGTT MAXENT: -18.01 MDD: -11.56 MM: -12.82 WMM: -13.06

TATTACTTCCCAGAGTATCTTTAACAGTATTCTCTGAAGCAGTTCCAATCTAGTTGGAGAATTAACAGCA MAXENT: -35.01 MDD: -26.21 MM: -27.05 WMM: -22.36

ATTGATTTAACTATCTCATTTTTATTAACTGTAATTTACTTTAAAAATATTTGCAAATCATACTCATTAG MAXENT: -33.33 MDD: -20.01 MM: -20.14 WMM: -14.78

TTATTTGATCATTGTTCTATGCATTTTAAAATTAATTTTGTGTTGTTCCTCTCAATATTTGTTTTTAACA MAXENT: -25.61 MDD: -16.33 MM: -15.34 WMM: -13.45

TTTATTCCCATTTTTATTTTATACTATTGTCTGTCATGCTTTATGTATTCCAATAAGTGTCTTGAAATCC MAXENT: -35.08 MDD: -21.40 MM: -21.10 WMM: -16.30

TTGTGGGGAAAGGCAGGACAAAAATAATTAGTTAATTAGATTTGAAAAATGTAATTTTTCCATTTTAAAT MAXENT: -15.24 MDD: -8.17 MM: -12.90 WMM: -12.25

ATTTCATTTGTATAAGAAAATATTTCAGAGAACCATGATGATAATGGATATGTGTGACTGTTTTGAATTT MAXENT: -21.38 MDD: -21.88 MM: -15.99 WMM: -16.56

TTTTCTCAATTAAAACATTTTGTATGTAA MAXENT: -45.04 MDD: -28.78 MM: -29.81 WMM: -24.11
